# Nomogram for the Therapeutic Efficacy of Apheresis Platelet Transfusion in Hematologic Patients

**DOI:** 10.1007/s12288-024-01857-0

**Published:** 2024-09-11

**Authors:** Yiwen He, Huihui Feng, Lu Yu, Gang Deng

**Affiliations:** 1 Department of Blood Transfusion, The First Affiliated Hospital of Ningbo University, Ningbo, Zhejiang 315010 China; 2 Institute of Blood Transfusion, Ningbo Blood Center, Ningbo, 315010 China

**Keywords:** Hematologic diseases, Nomogram, Platelet transfusion refractoriness, Prediction model

## Abstract

This study aims to explore the factors that affect the efficacy of apheresis platelet transfusion in patients with hematologic diseases and construct a nomogram prediction model to predict the possibility of obtaining satisfactory platelet transfusion efficacy and guide scientific and rational platelet transfusion. The basic information of 2,007 hematologic patients who received apheresis platelet transfusions from June 2022 to April 2023 and the corresponding donor information and apheresis platelet data are collected. The risk factors that cause ineffective platelet transfusions are screened through a logistic regression analysis. Then, the risk factors are introduced into R software, and a nomogram prediction model is established and validated. The regression analysis shows that the independent risk factors for ineffective platelet transfusion are platelet count before transfusion, white blood cell count, hemoglobin content and mean corpuscular hemoglobin, cumulative platelet transfusion times, platelet antibody positivity, fever, splenomegaly, graft-versus-host disease, bleeding, and platelet storage days. These factors are included in the nomogram, and the calibration curve for predicting transfusion efficiency reveals good consistency between the nomogram-predicted results and the actual observations. The area under the curve obtained through internal repeated sampling is 0.756. This study comprehensively assessed the risks associated with factors leading to ineffective platelet transfusion and successfully constructed and validated a nomogram prediction model. This model provides an important predictive tool for assessing the efficacy of platelet transfusion in patients with hematologic diseases, with the potential to guide scientific and rational platelet transfusion practices.

**Supplementary Information:**

The online version contains supplementary material available at 10.1007/s12288-024-01857-0.

## Introduction

Platelets are widely used to treat or prevent bleeding, especially in patients with hematologic diseases [1]. About 10 million patients receive platelet transfusions every year, and among them, up to 67% have hematologic diseases; furthermore, the number of transfusions increases annually [2, 3]. However, for patients who receive long-term platelet transfusions, there might be cases of platelet transfusion refractoriness(PTR), such as failure to improve clinical symptoms or inadequate increase in platelet count after two or more consecutive transfusions of sufficient amount [4, 5]. PTR has caused unnecessary waste of blood platelets, making the already tight supply of blood platelets even more in short supply [6]. PTR can be caused by non-immune and immune factors. Among them, non-immune factors, such as fever, bleeding, spleen enlargement, disseminated intravascular coagulation (DIC), graft-versus-host disease (GVHD, drugs( such as amphotericin, vancomycin, etc.), longer platelet storage time and poor platelet quality, are the dominant ones, accounting for approximately 80% [7–9], while the remaining 20% are caused by immune factors, include human leukocyte antigen (HLA) antibodies, platelet alloantibodies, ABO antibodies and drug-dependent autoantibody [10, 11]. Currently, most studies discuss the factors that affect the efficacy of platelet transfusions from the perspectives of patient factors and platelet product quality [9, 12–14]. However, only a few studies have analyzed if the relevant factors of blood donors affect the effectiveness of platelet transfusion. Therefore, this study incorporates patient, platelet product, and blood donor factors into its analysis to establish a predictive model of efficacy, predict the probability of achieving satisfactory transfusion effects in patients with hematologic diseases, reduce the occurrence of ineffective transfusions, and guide clinical platelet transfusion.

## Materials and Methods

### Study Design

This was a single-center, retrospective study initiated by the First Affiliated Hospital of Ningbo University between June 2022 to April 2023. Patients who met the following criteria were included: patients with hematologic diseases over 18 years old who received apheresis platelet transfusion. The patients were excluded from the study if they suffered from immune thrombocytopenia or their clinical data was incomplete.

### Patient Data

We conduct a thorough search for patient information via the transfusion information system and the hospital information system. The comprehensive data gathered encompasses patients’ general details, current medical conditions, past medical history, medication records within the past 24 h prior to transfusion, any complications encountered, and the results of blood routine tests conducted before and after infusion.

### Donor Data

The donor’s information can be traced back through a product identification number of the apheresis platelets transfused to the patient, and the data was provided by the Ningbo Blood Center. The storage time and treatment of platelet products, as well as the general information of blood donors, number of blood donations and platelet count (PLT) before blood donation were collected.

### Preparation of Platelets

All platelets used in this study were apheresis platelets from Ningbo Blood Center and collected with a Cobe Spectra Apheresis Machine (FRESENIUS KABI, COM.TED). Each bag of apheresis platelets was one therapeutic unit (PLT ≥ 2.5 × 10^11^ units; capacity, 250–300 mL; stored at 22 ± 2 ℃ after collection and preparation; and completed infusion within 5 days). Gamma (γ) irradiation was performed with cesium irradiators at doses of 25 Gy to 30 Gy. Blood typing re-examination and secondary blood matching were performed before transfusion.

### Indications for Platelet Transfusions

Preventive platelet transfusion was recommended when PLT was below or equal to 20 × 10^9^/L. When bleeding occurred, invasive procedures were needed or the patient’s life was in danger, the threshold value was appropriately increased.

### Response to Platelet Transfusions

PLT after transfusion is influenced by the number of platelets transfused and the patient’s blood volume. Therefore, PLT alone is not a reliable measure to assess the effectiveness of platelet transfusion. Instead, corrected count increment (CCI) and percent of platelet recovery are used to adjust for these factors and provide an accurate assessment of post-transfusion platelet responses [15]. CCI is computed as [(platelet count after infusion – platelet count before infusion) (10^9^)] × body surface area (m^2^)]/input total number of platelets (10^11^). The body surface area calculation formula (m^2^) is height (cm) × 0.0061 + weight (kg) × 0.0128–0.01529. The CCI efficacy criteria of 1 h CCI < 7.5–24 h CCI < 4.5 can be regarded as ineffective platelet transfusion. Before platelet transfusion in this study, blood samples were collected for platelet counting. Blood samples were collected again 20–24 h after platelet transfusion for platelet counting. Therefore, this study regarded CCI < 4.5 as ineffective infusion.

### Statistical Analysis

Data processing and statistical analysis were performed using SPSS 27.0. Count data were presented as numbers or percentages and analyzed using the chi-square test. Measured data that followed a normal distribution were presented as the mean ± standard deviation and analyzed using the independent-sample t-test. Measured data that did not follow a normal distribution were presented as the median (P25, P75) and analyzed using the Wilcoxon test. The risk factors were screened out by a logistic regression model. The R packages “rms,” “calibrate,” and “pROC” were used to construct the prognostic nomogram, plot calibration curves, and ROC curves, respectively. A *P*-value of less than 0.05 was considered statistically significant.

## Results

### Characteristics of the Patients

The cohort included 2,007 patients with hematologic diseases, and the mean age was 57.31 ± 14.270 years. A total of 1,019 patients (comprising 50.78% of the cohort) were male, while 988 were female. Notably, despite this gender disparity, there was a negligible difference in the effective rate of infusion between males and females. The overall infusion effectiveness stood at a commendable 73.34%, indicating a consistent therapeutic response across genders.Among all diseases, the largest number of patients suffer from AML (51.92%), followed by MDS (21.77%), and lymphoma (12.06%) was relatively the least common. Almost all patients experience a simultaneous reduction in platelet count, white blood cell count, and suffer from anemia. Overall, the presence of platelet antibodies was documented in 66 patients (3.29%). In total, 39.71% of patients had previously received more than 10 platelet transfusions. Patients in the ineffective group experienced a higher proportion of fever, GVHD, splenomegaly, bleeding, infection, and thrombus. There was no statistically significant difference in platelet product factors and donor factors between the two groups (*P*>0.05) Table 1.

**Table 1:** Patient, blood donor, and platelet transfusion characteristics

Characteristic	Effective group		Ineffective group		χ2 /t/Z		*P*
	(*N* = 1472)		(*N* = 535)				
**Patient**							
Age: Mean ± SD, years	57.77 ± 14.43		56.04 ± 13.76		-2.393		0.017
BMI	22.24 ± 3.17		22.51 ± 3.40		1.640		0.101
Sex							
Men	743(50.48%)		276(51.59%)		0.153		0.696
Women	729(49.52%)		259(48.41%)				
Diagnosis							
AML	780(52.99%)		262(48.97%)		16.621		<0.01
MDS	288(19.57%)		149(27.85%)				
Lymphoma	188(12.77%)		54(10.09%)				
Others	216(14.67%)		70(13.08%)				
WBC before transfusion,10^9^/L	1.71(0.60,3.85)		1.50(0.50,3.80)		1.630		0.103
PLT before transfusion,10^9^/L	12(6,17)		12(7,17)		0.725		0.469
Hb before transfusion, g/L	71.95 ± 16.96		67.70 ± 15.22		-5.492		<0.01
MCH before transfusion, pg	31.52 ± 3.47		31.83 ± 2.76		-2.710		<0.01
PLT after transfusion,10^9^/L	37(26,50)		11(6,17)		29.751		<0.01
Platelet antibodies							
Negative	1438(97.69%)		503(94.02%)		15.497		<0.01
Positive	34(2.31%)		32(5.98%)				
Coombs test							
Negative	1410(95.79%)		499(93.27%)		4.820		0.028
Positive	62(4.21%)		36(6.73%)				
No. of platelet transfusions, times							
≤10	930(63.27%)		278(51.96%)		20.651		<0.01
>10	540(36.73%)		257(48.04%)				
Fever							
No	1442(97.96%)		412(77.01%)		241.650		<0.01
Yes	30(2.04%)		123(22.99%)				
GVHD							
No	1447(98.30%)		503(94.02%)		24.556		<0.01
Yes	25(1.70%)		32(5.98%)				
Splenomegaly							
No	1467(99.66%)		507(94.77%)		55.126		<0.01
Yes	5(0.34%)		28(5.23%)				
Bleeding							
No	1279(86.89%)		427(79.81%)		14.859		<0.01
Yes	193(13.11%)		108(20.19%)				
Infection							
No	1369(93.00%)		471(88.04%)		12.039		<0.01
Yes	103(7.00%)		64(11.96%)				
Thrombus							
No	1467(99.66%)		529(98.88%)		3.083		0.080
Yes	5(0.34%)		6(1.12%)				
Hepatic disease							
No	1267(86.07%)		466(87.10%)		0.271		0.603
Yes	205(13.93%)		69(12.90%)				
Use of antibiotics							
No	1326(90.08%)		467(87.29%)		2.924		0.087
Yes	146(9.92%)		68(12.71%)				
Use of immunoglobulin							
No	1420(96.47%)		513(95.89%)		0.226		0.635
Yes	52(3.53%)		22(4.11%)				
Hematopoietic stem cell transplantation							
No	1020(69.29%)		368(68.79%)		0.027		0.870
Yes	452(30.71%)		167(31.21%)				
**Platelet**							
Platelet irradiation							
No	1155(78.46%)		401(74.95%)		2.579		0.108
Yes	317(21.54%)		134(25.05%)				
ABO compatible							
Compatible	1390(94.43%)		509(95.14%)		0.262		0.609
Not compatible	82(5.57%)		26(4.86%)				
Days of storage, days							
≤2	668(45.38%)		225(42.06%)		1.624		0.203
>2	804(54.62%)		310(57.94%)				
**Blood donors**							
Age: Mean ± SD, years	41.31 ± 10.55		41.20 ± 10.28		-0.208		0.836
Sex							
Men	1053(71.54%)		375(70.09%)		0.330		0.566
Women	419(28.46%)		160(29.91%)				
BMI	24.29 ± 2.70		24.49 ± 2.72		1.491		0.136
PLT	250.65 ± 50.76		252.23 ± 51.48		0.616		0.538
NO.of blood donations	30(10,66)		30(10,68)		0.244		0.807

*Note* AML: acute myelocytic leukemia; MDS: myelodysplastic syndromes; Others include aplastic anemia, lymphocytic leukemia, multiple myeloma, and chronic myelogenous leukemia; PLT: platelet count; WBC: white blood cell count; Hb: hemoglobin content; MCH: mean corpuscular hemoglobin; GVHD: graft-versus-host disease

### Independent Risk Factors in the Cohort

For the primary cohort, we conducted a univariate logistic regression analysis of each clinical factor (Table 2) and selected factors with a *P*-value less than 0.1. The factors of interest were also included in the multivariate logistic regression (Table 3). The variable assignments are presented in Table 4. The results showed that no significant relationship existed between donor-related factors and transfusion efficacy. However, the patient and platelet product variables were significantly associated with the outcomes of multivariable modeling, including PLT before transfusion, white blood cell (WBC) count before transfusion, hemoglobin content (Hb) before transfusion, mean corpuscular hemoglobin before transfusion, number of platelet transfusions, platelet antibodies, fever, splenomegaly, graft-versus-host disease (GVHD), bleeding at the time of the infusion, and days of platelet storage.

**Table 2:** Univariate logistic regression analysis

		OR(95%CI)		*P*
**Patient**				
Sex		1.05(0.86,1.27)		0.66
Age		1.01(1,1.02)		0.02
MDS		0.65(0.51,0.83)		＜0.01
Lymphoma		1.16(0.83,1.62)		0.39
others		1.05(0.77,1.42)		0.77
BMI		0.97(0.95,1.01)		0.10
PLT before transfusion,109/L		1(0.99,1.01)		0.82
WBC before transfusion,109/L		1.01(1,1.02)		0.01
Hb before transfusion, g/L		1.02(1.01,1.02)		＜0.01
MCH before transfusion, pg		1.05(1.01,1.09)		0.01
No. of Platelet transfusions		0.63(0.51,0.77)		＜0.01
Platelet antibodies (Ab)		0.37(0.23,0.61)		＜0.01
Coombs test		0.61(0.4,0.93)		0.02
Fever		0.07(0.05,0.11)		＜0.01
Splenomegaly		0.06(0.02,0.16)		＜0.01
GVHD		0.27(0.16,0.46)		＜0.01
Infection		0.55(0.4,0.77)		＜0.01
Bleeding		0.6(0.46,0.77)		＜0.01
Thrombus		0.3(0.09,0.99)		0.05
Hepatic disease		1.09(0.82,1.46)		0.55
Use of antibiotics		0.76(0.56,1.03)		0.07
Use of immunoglobulin		0.85(0.51,1.42)		0.54
Hematopoietic stem cell transplantation		0.98(0.79,1.21)		0.83
**Platelet**				
Days of storage		0.87(0.72,1.07)		0.19
Platelet irradiation		0.82(0.65,1.04)		0.10
ABO compatible		0.87(0.55,1.36)		0.53
**Blood donors**				
Sex		0.93(0.75,1.16)		0.53
Age		1(0.99,1.01)		0.84
BMI		0.97(0.94,1.01)		0.14
PLT		1(1,1)		0.54
No.of Platelet donations		1(1,1)		0.92

**Table 3:** Multivariate logistic regression analysis

		OR(95%CI)		*P*
**Patient**				
Age		1(0.99,1.01)		0.36
MDS		0.79(0.56,1.11)		0.17
Lymphoma		0.95(0.63,1.45)		0.83
Others		1.06(0.73,1.56)		0.75
BMI		0.96(0.92,1)		0.06
PLT before transfusion,109/L		0.98(0.97,0.99)		0.01
WBC before transfusion,109/L		1.01(1,1.02)		0.01
Hb before transfusion, g/L		1.01(1.01,1.02)		＜0.01
MCH before transfusion, pg		1.07(1.01,1.14)		0.02
No. of platelet transfusions		0.65(0.49,0.85)		＜0.01
Platelet antibodies (Ab)		0.49(0.27,0.89)		0.02
Coombs test		0.68(0.39,1.17)		0.16
Fever		0.1(0.06,0.16)		＜0.01
Splenomegaly		0.05(0.02,0.14)		＜0.01
GVHD		0.26(0.11,0.58)		＜0.01
Infection		0.92(0.54,1.57)		0.76
Bleeding		0.62(0.43,0.9)		0.01
Thrombus		0.39(0.1,1.48)		0.17
Use of antibiotics		1.22(0.83,1.77)		0.31
**Platelet**				
Days of storage		0.74(0.57,0.96)		0.03
Platelet irradiation		1.1(0.75,1.61)		0.62

**Table 4:** Logistic regression assignment

Characteristic		assignment
**Patient**		
Sex		Men = 0,Women = 1
Diagnosis		
AML		1
MDS		2
Lymphoma		3
Others		4
No. of platelet transfusions, times		≤10 = 0, >10 = 1
Platelet antibodies (Ab)		Negative = 0, Positive = 1
Coombs test		Negative = 0, Positive = 1
Fever		No = 0, Yes = 1
Splenomegaly		No = 0, Yes = 1
GVHD		No = 0, Yes = 1
Infection		No = 0, Yes = 1
Bleeding		No = 0, Yes = 1
Thrombus		No = 0, Yes = 1
Hepatic disease		No = 0, Yes = 1
Use of antibiotics		No = 0, Yes = 1
Use of immunoglobulin		No = 0, Yes = 1
Hematopoietic stem cell transplantation		No = 0, Yes = 1
**Platelet**		
Days of storage, days		≤2 = 0, >2 = 1
Platelet irradiation		No = 0, Yes = 1
ABO compatible		No = 0, Yes = 1

### Prognostic Nomogram for the Probability of Effective Platelet Transfusion

**Fig. 1:**
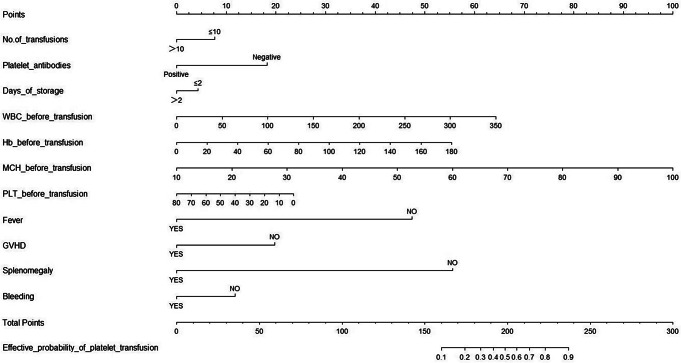
Nomogram for predicting platelet transfusion efficacy

**Fig. 2:**
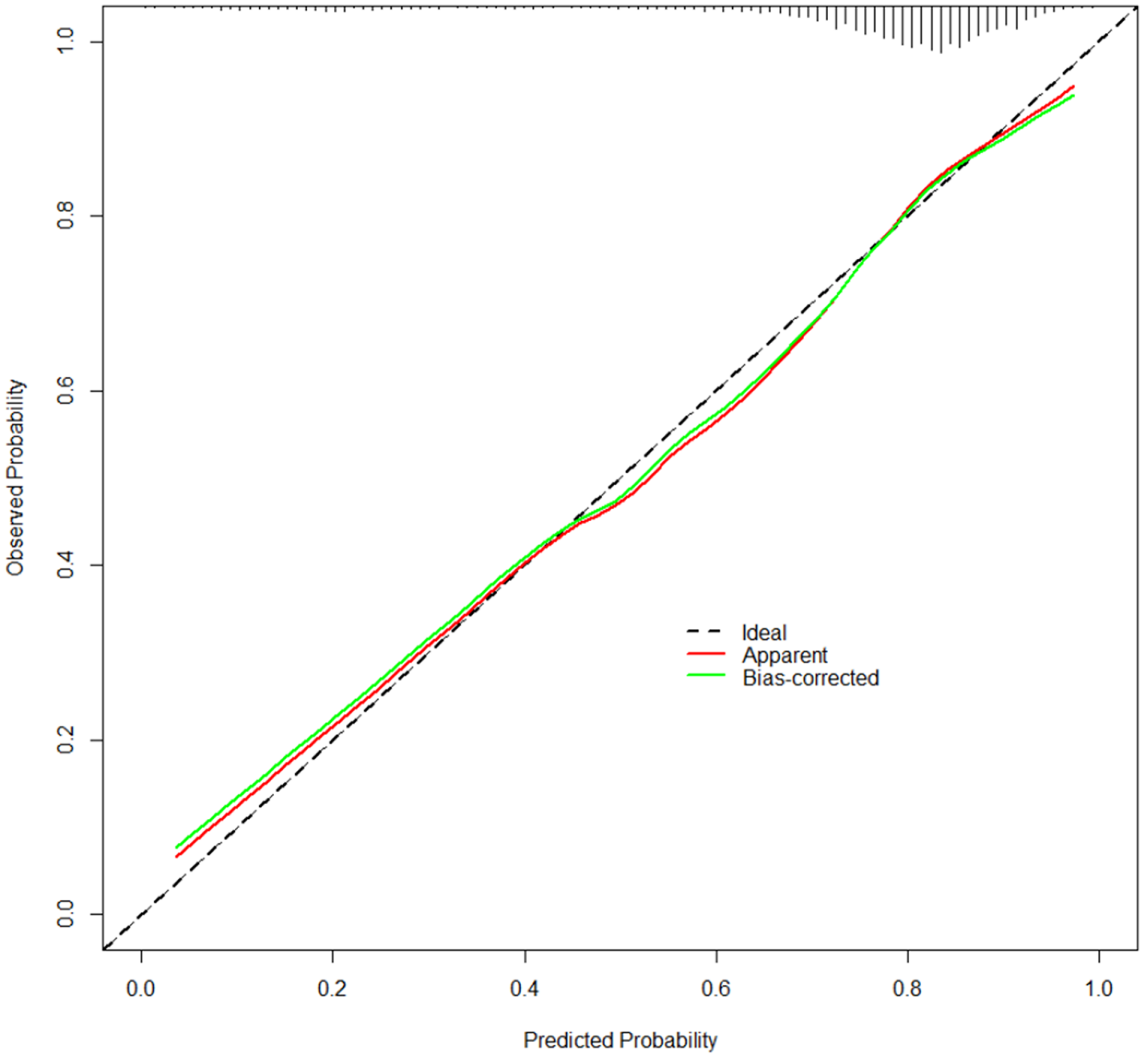
Calibration curve for the nomogram. The nomogram-predicted probability is plotted on the x axis; the actual probability is plotted on the y axis

A prognostic nomogram that integrates all significant independent factors for infusion efficacy in the primary cohort is shown in Fig. 1. The nomogram indicates that the blood indicators prior to transfusion considerably affected the outcome. Notably, effective infusion can be particularly challenging for patients experiencing a fever, splenomegaly, and GVHD. A total score was obtained by assigning a score to each variable and adding each item score. A high score indicated a high probability of receiving an effective infusion. The calibration plot for the probability of effective infusion showed optimal agreement between the nomogram predictions and the actual observations (Fig. 2).

### ROC for Predicting Platelet Transfusion Efficacy

**Fig. 3:**
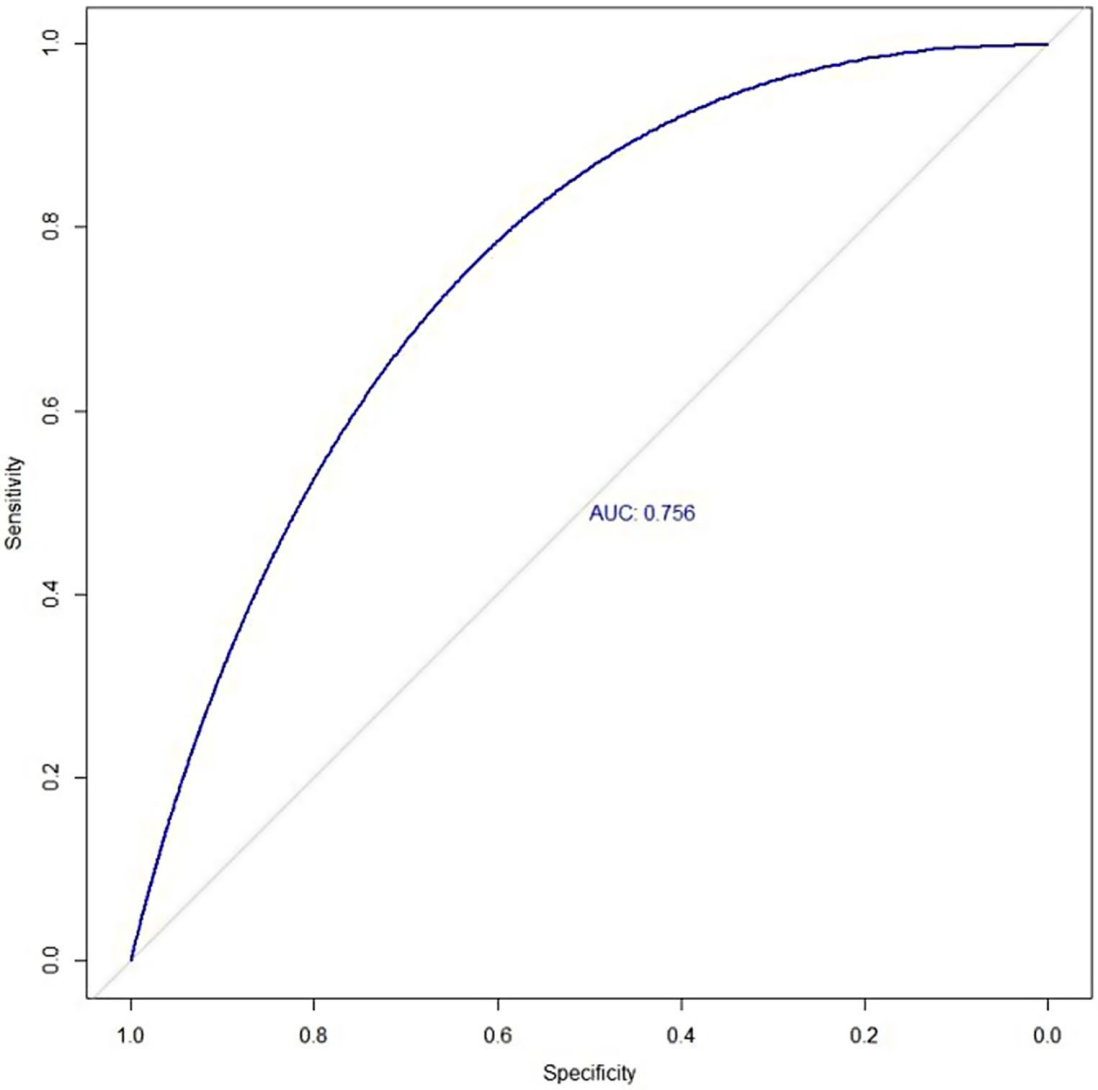
Receiver operator characteristic curves for predicting platelet transfusion efficacy. AUC refers to the area under the receiver operator characteristic curve

The area under the receiver operating characteristic curve was 0.756 (Fig. 3).

## Discussion

The hematopoietic system in the bone marrow of patients with blood disease is suppressed or even destroyed due to disease reasons, and platelet production and maturation are hindered. These patients usually need to receive repeated platelet transfusions to prevent or treat bleeding [16]. However, not every infusion can achieve satisfactory results. Both immunological and non-immunological factors contribute to the occurrence of PTR [17]. Moreover, individual variations significantly impact the effectiveness of platelet transfusion outcomes. In current study, the immunological factor is platelet antibody, and other factors are all non-immunological factors.

Logistic regression analysis found that the age, body mass index, pre-donation platelet count, and number of platelet donations of blood donors had no significant effect on the effectiveness of patient transfusion. Numerous studies have consistently demonstrated an elevated risk of mortality in patients receiving red blood cells donated by female blood donors. However, in the realm of platelet transfusion, no research has identified gender as a factor that contributes to an increased risk of adverse events [18–20]. Consistent with these findings, our study likewise did not uncover any significant influence of gender on the effectiveness of platelet transfusion. It may be that we strictly screen blood donors during platelet collection, and the collection process is strictly implemented according to requirements. Each bag of apheresis platelets meets the requirements for clinical infusion. Although the factors of blood donors currently included in the analysis have no significant impact on the infusion effect, it cannot be confirmed whether other factors, such as the lifestyle, pregnancy history and medication history of blood donors, will affect the infusion effect. This remains to be further investigated and analyzed. The extended storage time of platelets will reduce the efficiency of infusion. Studies have shown that the extended storage time will reduce the number and aggregation function of platelets, while increasing the release of inflammatory factors, especially after the third day, resulting in a decrease in the efficiency of platelet infusion [9, 21, 22]. Therefore, we recommend that for patients with poor conditions and requiring long-term multiple platelet transfusions, try to give them more fresh platelets.

The blood indicators before platelet transfusion considerably affect transfusion efficacy. Patients with near-normal WBC counts and Hb before transfusion have a mild condition, and the destruction of bone marrow hematopoiesis is minimal, which increases the effectiveness of transfusion. However, excessive PLTs before transfusion can reduce the efficiency of transfusion, so the threshold for platelet transfusion must be controlled when PLTs are too high to avoid preventive transfusion. An increase in the body mass index and blood volume of patients reduces the increase in PLTs after transfusion [15]. Therefore, patients should control their weight to prevent excessive blood volume from weakening the infusion effect.

In agreement with existing data, our data indicate a high risk of poor PLT increment associated with the presence of platelet antibodies and increased numbers of transfusions [13]. We found that patients who had myelodysplastic syndrome had a higher risk of refractoriness to platelet transfusion compared with patients who had acute leukemia [23, 24]. However, after controlling for confounding factors, the multivariable logistic regression suggested that myelodysplastic syndrome did not considerably affect the efficacy of platelet transfusion. However, when patients are transfused with platelets, the effectiveness of the transfusion will be greatly reduced if they have a fever, GVHD, splenomegaly, and bleeding. The rise in body temperature due to fever stimulates an increase in the release of inflammatory factors and tumor necrosis factors. Additionally, this elevation in temperature facilitates blood circulation, which subsequently accelerates the destruction of platelets by the mononuclear macrophage system [14]. Changes in the spleen cause platelets to be retained, concurrently augmenting the phagocytic activity of macrophages, ultimately leading to an increased destruction of platelets. Therefore, patients should control their body temperature before receiving platelet transfusions, and platelet transfusions should not be performed on patients with a high fever unless the case is an emergency. We recommend splenectomy for patients with spleen enlargement and surgical indications. Platelets after hematopoietic stem cell transplantation and irradiation do not reduce the efficiency of transfusion.

The nomogram is a commonly used tool in medical research. It integrates different factors on the basis of clinical characteristics, making the regression analysis visual and intuitive in revealing the effects of different factors on outcomes [25–27]. Although all the factors in the nomogram model influence the infusion effect, the nomogram we constructed showes the state of platelet infusion exerts a much greater effect on the infusion than the number of platelet infusions and the storage days of platelet products. Current research on the efficacy of platelet transfusion focuses on analyzing the influencing factors through transfusion efficacy, and models for predicting outcomes are lacking. In this study, we obtained the independent risk factors that affect transfusion efficacy through logistic regression analysis. With the help of R software, we established a predictive nomogram model that combines multiple clinical factors to quantitatively calculate the probability of obtaining effective transfusion for patients, which can help clinicians implement measures in advance.

The AUC of the predictive model constructed by R software in this study was 0.756, and the calibration curve obtained through internal validation was similar to the real one, indicating that the model has good diagnostic value. The limitation of this study is the lack of external validation. In the future, cooperation with other medical centers should be established to prevent model overfitting and obtain an accurate predictive model, thereby further improving the efficiency of platelet transfusion.

## Electronic Supplementary Material

Below is the link to the electronic supplementary material.

Supplementary Material 1
