# Supplementary material for: Nomogram for the Therapeutic Efficacy of Apheresis Platelet Transfusion in Hematologic Patients
Source: Indian J Hematol Blood Transfus. 2024 Sep 11;41(3):656–64. doi: 10.1007/s12288-024-01857-0 (PMC12267762; doi:10.1007/s12288-024-01857-0)
Supplement: Supplementary file 1 — Supplementary Material 1 [file 12288_2024_1857_MOESM1_ESM.docx]

**涉及人的生物医学研究伦理审查批件**

**Ethics** **Committee** **Approval** **Letter** **of** **Biomedical** **Research** **Involving** **Humans**

**批件号Approval** **NO.：** 宁波大学附属第一医院伦审2023研第200RS号

| 项目名称  Study Title | 影响血液病患者单采血小板输注效果的相关因素分析 | | |
| --- | --- | --- | --- |
| 申办方  Sponsor | / | | |
| 受理号  Acceptance Number | 宁波大学附属第一医院伦审2023研第200RS号-01 | | |
| 主要研究者  Principal  Investigator | 何意文 | 承担科室  Responsible  Department | 输血科 |
| 审查类别  Category of Review | 初始审查 | 审查方式  Type of Review | 简易审查 |
| 审查日期  Date of Review | 2024年01月02日 | 审查地点  Location of  Review | 宁波大学附属第一医院伦 理办公室 |
| 审查文件清单  Items Reviewed | 临床研究方案（1.0，2023.12.28） .pdf(1.0,2023-12-28)  临床研究方案（1.0，2023.12.28） .pdf(1.0,2023-12-28)、免签知情同意书 .pdf(1.0,2023-12-28)  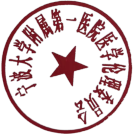初始审查申请表.pdf、科学性审查批件.pdf、研究者履历表.pdf、研究者责任声 明.pdf、研究者利益冲突声明.pdf | | |
| 审评意见  Evaluation | 同意 | | |
| 审查决定  Decision | 委员会对该项目的审查决定为： ■同意 (Approval) | | |
| 主任/副主任委员  签字  Chair Signature | 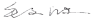 | | |
| 签发日期  Date of issue | 2024年01月03日 | | |
| 伦理审查委员会  Stamp of EC | 伦理审查委员会盖章 | | |
| 批件有效期  Period of Validity | 自本伦理审查委员会初始审查批准之日起一年内，本临床研究应在本院启动。逾 期未启动的，本批件自行废止。 | | |
| 年度/定期跟踪审查  Continue Review | 审查频率为该研究批准之日起每 12 月一次，首次，请于 2025年01月03日 前 1个月递交研究进展报告。  伦理审查委员会有根据实际进展情况改变跟踪审查频率的权利。 | | |
| 声明  Statement | 本伦理审查委员会的职责、人员组成、操作程序及记录遵循《涉及人的生物医学 研究伦理审查办法》、《涉及人的健康相关研究国际伦理准则》、《赫尔辛基宣 言》、GCP和ICH-GCP等国际伦理指南和国内相关法律法规。 | | |

宁波市海曙区柳汀街59号 邮编：3150101

1 联系人：陈少莹 电话：0574-87085233

| **注意事项：**  1.请遵循我国相关法律、法规和规章中的伦理原则。  2.请遵循经本伦理审查委员会批准的临床研究方案、知情同意书、招募材料等开展本研究，保护受试者的健 康与权利。对研究方案、知情同意书和招募材料等的任何修改，均须得到本伦理审查委员会审查同意后方可 实施。  3.在本院发生的SAE/SUSAR以及研发期间安全性更新报告须按照NMPA/GCP最新要求及时递交本伦理审查委员 会，国内外其它中心发生的SAE/SUSAR需定期汇总、评估后递交本伦理审查委员会。  4.根据报告情况，本伦理审查委员会有权对其评估做出新的决定。  5.自今日起，无论研究开始与否，请在跟踪审查日到期前1个月提交研究进展报告。  6.申办方应当向组长单位伦理审查委员会提交中心研究进展报告汇总；当出现任何可能显著影响研究进行或 增加受试者危险的情况时，请申请人及时向本伦理审查委员会提交书面报告。  7.研究纳入了不符合纳入标准或符合排除标准的受试者，符合中止研究规定而未让受试者退出研究，给予错 误治疗或剂量，给予方案禁止的合并用药等没有遵从方案开展研究的情况；或可能对受试者的权益或健康以 及研究的科学性造成不良影响等违背GCP原则的情况，请申办方、监查员或研究者提交违背方案报告。  8.申请人暂停或提前终止临床研究，请及时提交暂停或终止研究报告。  9.完成临床研究，请申请人提交结题报告。  10.凡涉及中国人类遗传资源采集标本、收集数据等研究项目，必须获得中国人类遗传资源管理办公室批准 后方可在本中心开展研究。  11.凡经本伦理审查委员会批准的研究项目在实施前，申请人应按相关规定在国家卫健委、药审中心等的临 床研究登记备案信息系统平台登记研究项目相关信息。 |
| --- |

宁波市海曙区柳汀街59号 邮编：3150101

2 联系人：陈少莹 电话：0574-87085233
